# German Cat and Dog Owners’ Views on Veterinary Error Handling: Communication and Transparency Concerns from Qualitative Interviews

**DOI:** 10.3390/ani15202981

**Published:** 2025-10-15

**Authors:** Vivian K. Johann, Claudia Busse, Holger A. Volk, Christin Kleinsorgen

**Affiliations:** 1 Department of Small Animals Medicine and Surgery, University of Veterinary Medicine Hannover, 30559 Hannover, Germany; 2 Centre for Teaching, University of Veterinary Medicine Hannover, 30559 Hannover, Germany

**Keywords:** critical incidents, medical error culture, medical mistakes, veterinarians, professionalism, pet owner

## Abstract

Medical errors in veterinary practice have a significant impact on veterinary staff, but the emotional experiences of pet owners remain largely unexplored. A total of 23 German pet owners participated in this qualitative study, which explored the impact of medical errors on their expectations of veterinary surgeons through semi-structured online interviews conducted between September and November 2024. The interviews followed a pretested questionnaire and were subsequently subjected to qualitative analysis. They reported emotional distress when faced with a medical error, characterised by anxiety, worry and helplessness. This led to increased scepticism and caution towards other veterinary surgeons, as well as a decline in trust and satisfaction with veterinary care. Pet owners emphasised the importance of veterinary surgeons being open and honest about errors, communicating openly and working together to prevent similar incidents. Pet owners are concerned about inadequate communication, lack of transparency and insufficient empathy from veterinary surgeons in the event of an error. Based on that, enhancing empathetic communication and providing comprehensive information could minimise the potential for conflict. Ensuring that pet owners feel acknowledged and respected is essential for fostering trust and long-term professional relationships.

## Simple Summary

This manuscript explores the experiences of dog and cat owners in Germany with errors in veterinary medicine. Through 23 semi-structured individual interviews, the subjective perspectives of the pet owners were elicited and analysed. The findings reveal that pet owners often feel helpless, uncertain and anxious when they perceive an error. They desire open and honest communication, where their concerns and anxieties are taken seriously and solutions are sought collectively. The study demonstrates that errors in veterinary medicine not only affect the animals and veterinary staff, but also the pet owners. The results also show that honesty, empathy and adequate communication are crucial in addressing errors.

## 1. Introduction

Animals are regarded as an integral part of the family or a partner by many people [1,2,3]. Given this close relationship, it is not unexpected that pet owners have high expectations for veterinary care, increasingly comparing it to the standards available in human medicine [4]. Clear and effective communication is essential in meeting these expectations, as it allows veterinary surgeons to provide the best possible care and involves pet owners in the decision-making process [5,6,7]. Effective communication is a cornerstone of high-quality veterinary care [8]. Conversely, negative experiences in veterinary care are often attributed to poor communication [9].

A study from Turkey retrospectively examined complaints from animal owners [10]. It identified that clinical issues were mainly due to medical errors and substandard care, while relationship issues were primarily driven by a lack of communication and insufficient information [10].

Medical errors reported by the veterinary team itself in a reporting system were mainly related to medicines (>54%) and communication (>21%) [11]. These findings highlight the need for improved communication and error management in veterinary care, which is a critical aspect of providing high-quality care.

Despite recent advances in veterinary medicine, errors still occur [11,12,13,14]. Making mistakes is human [15]. A single error can have far-reaching consequences not only for the affected individual (pet owner or animal) but also for the person responsible [16].

Studies have also shown that near misses and medical errors in veterinary medicine can have a negative impact on the mental health of veterinary surgeons. Negative effects include reduced job satisfaction, reduced self-confidence, shame and sleep disturbances [13,17,18].

Structured reporting systems and clear feedback mechanisms can facilitate the disclosure of errors by veterinary surgeons [15,19,20]. By adopting similar systems, veterinary medicine can strengthen its error culture and improve patient safety.

It is therefore crucial to learn from errors and implement preventive strategies.

While several studies have explored the owner’s role in everyday consultations, communication and the delivery of bad news [1,5,7,21], research has mostly focused on the veterinarian’s perspective when errors occur [22].

Despite the frequent occurrence of medical errors in veterinary medicine, there is still limited research on how pet owners experience and perceive these events. It is not yet clear what dog and cat owners in Germany perceive as errors in veterinary medicine. Therefore, this study aims to provide initial insights into owners’ perception of error, particularly regarding their expectations with regard to veterinary surgeons in Germany in handling these situations.

## 2. Participants and Methods

### 2.1. Study Design

Semi-structured individual online interviews were used to collect data. These were conducted in German language. A qualitative study enabled a more personal trustworthy relationship to be established with the participants, providing a deeper understanding of their needs [23].

### 2.2. Participants

Participants were invited to participate voluntarily via the social media platforms Facebook^®^ (pet owner groups) and Instagram^®^ as a non-probabilistic, independent quota sample of participants. It was also posted on the “Fehlerkultur Tiermedizin” account. A convenience sampling approach was used. Recruitment via social media may have biased the sample towards individuals active on these platforms, potentially excluding those without access or less comfortable with digital communication.

Inclusion criteria for participation were as follows: participants had to be at least 18 years old, proficient in the German language and have experience as current or former pet owners of dogs and/or cats and have experienced an error in veterinary medicine.

### 2.3. Interview Guide

The interview guide consisted of 40 questions, with 23 open-ended and 17 closed questions (Supplementary material: Table S1). The questions were developed based on literature and discussion within the project team. The questionnaire was influenced by the research of Coe, Adams [24], Cipolla et al. [1] and Powell et al. [25], whose work contributed to questions about the experiences and wishes of pet owners when dealing with errors. The publication by White [22] helped to develop questions about the feelings of pet owners and their need to talk about them. The first section collected personal data and experiences as pet owners and with veterinary surgeons. The next set of questions examined the views of animal owners on errors in veterinary medicine, including the type of errors perceived, expectations and needs. The next section focused on conversations between pet owners and veterinary surgeons in the event of an error, including treatment options, alternatives, risks and costs. The final section assessed the consequences of the error from the pet owners’ perspective, including their awareness of mistakes in veterinary medicine. A final open-ended question allowed participants to add any additional comments or insights regarding the topic of errors in veterinary medicine.

### 2.4. Pre-Interviews

Three pre-test interviews were conducted prior to the main study. These pre-tests aimed to review and, if necessary, revise the questionnaire as well as provide an initial estimate of the time required for the interviews. The pre-tests also served as preparation for the interviewer to conduct the interviews with the participants beforehand [26,27]. The data from pre-test interviewees were not included in the final analysis.

### 2.5. Main Interviews

This study aimed to collect subjective accounts of events, rather than to track or evaluate errors. The term “error” was entirely defined by the participants, with no verification or review conducted to assess whether the care provided adhered to established medical standards. Furthermore, the perceived veterinary errors could not be verified for plausibility, as the situations described were not evaluated from a legal or veterinary perspective. If participants had already answered questions on their own initiative, these were not asked again, unless they served to improve the interviewer’s understanding. Some follow-up questions became redundant once the previous ones had been answered and were therefore not asked. The duration of an interview was dependent on the interviewee’s narrative flow of storytelling needs and was not precisely determined in advance. The median duration of an interview was 32 min (shortest: 16 min, longest: 90 min).

### 2.6. Transcription

The interviews were conducted, transcribed and analysed by one person, with supervision from the co-authors, between September 2024 and November 2024. For processing and analysing the data, the word processing software Microsoft Word^®^ (version 16.100.1 (25081721)), the audio and video recordings from Microsoft Teams^®^ (version 25255.703.3981.5698) and the spreadsheet software Microsoft Excel^®^ (version 16.101.1 (25092124)) were used.

The interviews were conducted online via Microsoft Teams^®^, recorded and transcribed. The spoken word was transcribed verbatim. The transcripts were postedited. To ensure the study’s focus on thematic content, non-verbal cues and secondary verbal expressions were carefully removed from the transcripts. Additionally, any unintentional repetition, filler words or sounds and minor speech errors were corrected or omitted [28]. Also, raw data were anonymised.

### 2.7. Data Analysis

The primary investigator was supervised by coauthors who are trained in qualitative methods. Also, literature from the social sciences, as well as literature on qualitative studies, were used for training [23,29]. The analysis aimed to represent owners’ perspectives naturally and objectively [30].

The qualitative content analysis by Mayring [30] was applied to the analysis of the interviews, which aligns with the thematic 6-step approach by Braun and Clark [31]. Following a series of repeated readings of the transcripts and familiarisation with the contents (step 1—familiarisation), individual interview passages were then paraphrased and generalised to a higher level of abstraction as categories (codes) by one researcher (step 2—generation of initial codes). Within the next iterative steps (3—search, 4—review and 5—definition of codes), the transcripts were repeatedly reviewed.

The categories were formed through multiple readings and analyses of the interviews. The recurring codes and aspects were identified and grouped into categories. For the purposes of conceptualisation, perceived errors, individual emotions, perceived reactions, desired responses, and consequences were grouped into thematic categories. These categories are presented in the results and provide an overview of the key themes and aspects that emerged from the interviews. All cases described could be assigned to at least one category. Any uncertainties in the categorisation were clarified through discussion among the co-authors, and a consensus was reached. The inductive thematic analysis was used to form categories directly from the research material. These categories were not pre-defined but emerged from the analysis of the data. Similar passages were grouped into overarching units of meaning, taking into account both linguistic and conceptual nuances. The interpretation aimed to identify the central meanings and recurring patterns within participants’ statements. Interim findings were additionally discussed within the research team to reflect on alternative interpretations and to ensure the plausibility of the analytical conclusions.

The key statements which responded to the research questions were transferred to an Excel^®^ spreadsheet.

This manuscript contains only a descriptive evaluation (step 6—create a report). The collection of data as well as the report writing also followed the COREQ checklist [32]. Descriptive summaries were used to present findings, highlighting general frequency patterns, with selected qualitative and closed-question data quantified (e.g., participant characteristics, animal species, veterinarian type, trust, and owner–veterinarian relationship). Multiple responses were analysed dichotomously, and percentages rounded.

Chat AI from Academic Cloud was used to formulate some sentences more comprehensibly [33], DeepL Write AI writing assistant was used to translate sections of the manuscript [34] and interviews were transcribed using Microsoft Copilot [35]. The manuscript was translated after the description and analysis had been completed. The content was reviewed by the authors.

### 2.8. Ethics Statement

This study was conducted in compliance with the ethical standards of the University of Veterinary Medicine Hannover, Foundation. The Ethics Committee of the university approved the project in accordance with ethical guidelines for research involving human participants. Furthermore, the university’s Data Protection Officer granted approval for the project. All participants voluntarily consented to the processing of their data in accordance with the General Data Protection Regulation (GDPR) (2018) of the European Union (EU), specifically Article 6(1)(e) in conjunction with Article 89, as well as the Lower Saxony Data Protection Act (§ 3(1) No. 1 NHG, § 13). Data processing was carried out anonymously in line with the university’s Data Protection Regulation. All collected data were anonymised or pseudonymised for analysis.

All participants provided prior consent for data processing, except for one participant who declined a video recording. This person was interviewed with an audio recording alone.

## 3. Results

The following results have been divided into different sections to provide a clearer overview. First, the socio-demographic data is listed (Section 3.1). This is followed by a second outlining the errors perceived in veterinary medicine (Section 3.2). Thereafter, the emotions and reactions of the animal owners in relation to the event are described (Section 3.3). The following section then examines the extent to which there was any discussion of the perceived error (Section 3.4). The final section then presents a summary of the consequences of the event for the animal owners (Section 3.5).

### 3.1. Personal Data and Experience of Pet Ownership

**Figure 1:**
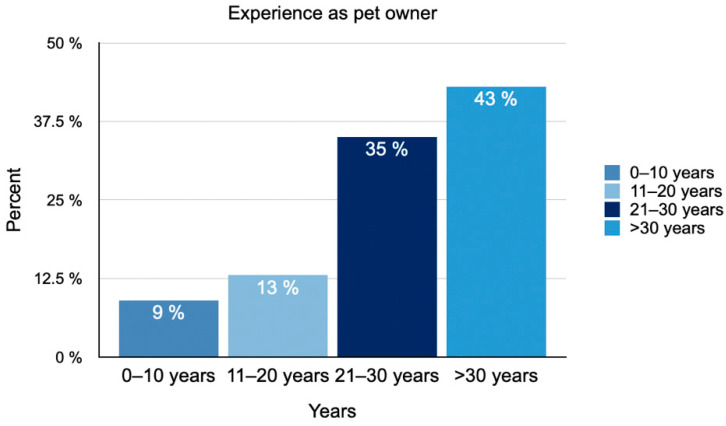
Distribution of pet owners’ experience with animals, measured in years (N = 23).

A total of 23 participants took part in the study. Of the participants, 74% identified themselves as female and 26% as male. The age of the participants at the time of the survey ranged from 25 to 63 years (median: 47 years). Thirty percent of the participants had some medical knowledge. Medical knowledge refers to the expertise acquired through formal education and training in healthcare professions such as nursing, pharmacology, medicine, veterinary nursing and related fields. The experience as a pet owner ranged from 8 to over 50 years (median: 26 years) (Figure 1).

**Figure 2:**
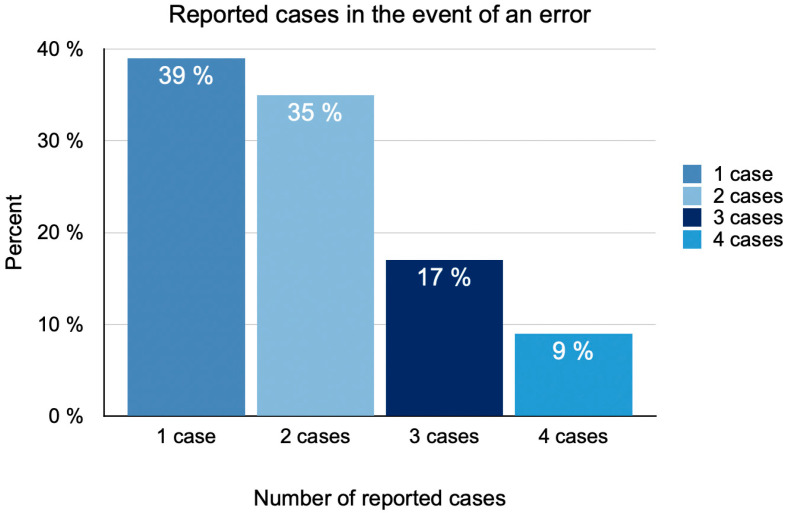
The diagram illustrates the number of error reports received from pet owners (N = 23).

Ninety-one percent of the participants stated that their pets were under the care of one General Veterinary Practitioner at the time of the interviews. In total, 45 cases of errors by veterinary surgeons were reported in the interviews (Figure 2). On average, each participant described two cases. In 76% of the cases, dogs were involved, while in 24% of the cases, cats were involved.

The cases reported by the participants occurred at a median of 4 years previously (range: same year as interview to over 20 years previously). Approximately half of the reported incidents occurred under the care of the participants’ General Veterinary Practitioner (49%). Almost every fourth case (24%) happened in a veterinary practice besides that of their General Veterinary Practitioner. One in five errors occurred in an emergency practice and a small number of events happened in clinics (4%) or other institutes, such as pathology (2%).

### 3.2. Pet Owners’ Perspectives on Errors

A total of 45 cases concerning errors in veterinary medicine were reported in the interviews. The reported errors were categorised into six types.

#### 3.2.1. Diagnosis Related

The majority of reported errors were related to diagnosis or diagnostics. According to one participant: “My dog was very tired and exhausted and could hardly go for longer walks. I went to the veterinarian, who rather quickly diagnosed a thyroid problem and prescribed thyroid medication. We tried it, and she was given the medication, but it only improved her condition by about 5%, so hardly noticeably. Then the idea was to increase the dosage, but I thought that did not seem right. In the end, it turned out that the dog did not suffer from a thyroid condition at all.” [Interview 10, Case 23].

#### 3.2.2. Interaction with Pet Owners

The analysis revealed shortcomings in the handling of pet owners, characterised by both a lack of empathy and inadequate communication. Some owners reported feeling dismissed or not taken seriously by veterinary surgeons. For example, one participant said: “The veterinarian’s wife practically dismissed me rather brusquely at the window and, without even looking at my animal or asking further questions, simply said that the practice would reopen later.” [Interview 5, Case 10].

Additionally, there were reports of surgical methods not being agreed upon, with one owner stating: “I discussed the procedure again with the veterinary surgeon, who explained that she would only approach between the toes from above and examine everything; operating on the pad was not planned. After the procedure, she stated that she had delegated the operation to a colleague, having previously discussed all relevant points. Nevertheless, the pad had been cut.” [Interview 2, Case 6].

Moreover, concerns were raised about the veterinary surgeons’ approach, which was perceived as arrogant and disrespectful. An owner reported: “We really felt—I don’t even know how to describe it—it was truly disrespectful, not properly acknowledged.” [Interview 22, Case 43].

#### 3.2.3. Handling of Animals

Several participants expressed concerns regarding the handling of their animals. The lack of appreciation for the emotional bond between animal and owner was perceived as problematic. For instance, one owner reported: “In autumn, my dog was tied to a freezing cold radiator in the practice.” [Interview 2; case 4]. In addition, one participant reported: “The veterinarian hit the cat, which had become rather resistant, then wrapped her in a cloth and pulled her around.” [Interview 23, Case 45].

#### 3.2.4. Treatment Related

A few owners perceived errors on the part of their veterinary surgeons was the treatment of their animals. For instance, one owner reported: “We went to the eye specialist, who examined our dogs eyes and the specialist said that it was a method used by the other vet, which is no longer used.” [Interview 17, Case 36]. In addition, in the case of a cat with ear mites, no further diagnostics were performed after no improvement had been seen, but the therapy was simply continued, which was later found out to be incorrect. Consequently, the animals were treated with an inappropriate therapy.

#### 3.2.5. Information Related

Some owners reported that they were not adequately informed about the treatment of their animals. The participants expressed concerns that they had not been adequately informed by the veterinary surgeons about the nature of the treatment, the potential side effects of medications, or the possibility of complications during surgery. For instance, one pet owner said: “We would have liked to have been informed that the vaccination might no longer be effective when combined with the medication.” [Interview 22, case 42].

#### 3.2.6. Anaesthesia Related

A few interviewees also encountered errors in connection with previous anaesthesia administered to their animals. They reported that their animals had woken up after anaesthesia with no medical staff present to monitor patients or that they had been returned to them weak and unresponsive after anaesthesia.

### 3.3. Emotions of the Pet Owners in the Event of an Error

**Figure 3:**
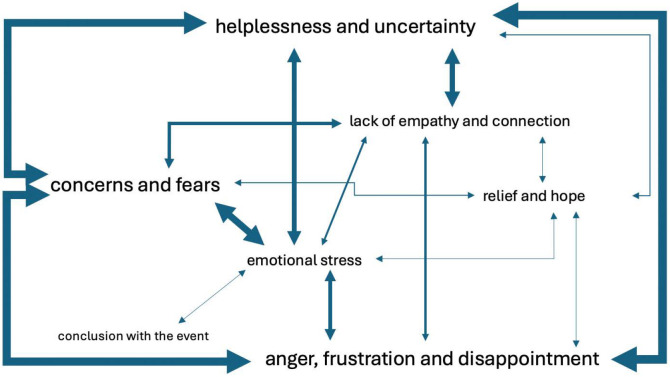
Concept map illustrating emotions identified in pet owners’ interviews concerning an error in veterinary medicine. The size of each label reflects how often that emotion was mentioned—larger labels indicate higher frequency. The thickness of the connecting lines represents how frequently two emotion categories were mentioned together—thicker lines denote a stronger co-occurrence.

The event of an error presented different emotional challenges for pet owners. The most common emotions of pet owners in the event of an error were categorised. The concept map presents a summary of inductively formed emotion categories and their connections to one another (Figure 3). The most frequently mentioned emotions, including those mentioned in conjunction with the others, were helplessness and uncertainty, concerns and fears, and anger, frustration and disappointment (Figure 3). Emotions like relief and hope and conclusion with the event were mentioned less frequently.

#### 3.3.1. Helplessness and Uncertainty

Many pet owners described feelings of helplessness and insecurity. They felt dependent on veterinary surgeons, relying on them for support and guidance regarding the care of their animals. As one owner explained: “You feel somewhat reliant on this person [the veterinary surgeon] to get clarity. […] ” [Interview 2, Case 3].

#### 3.3.2. Concerns and Fears

Several participants expressed that their concerns were not taken seriously, even after repeatedly raising them. One owner stated: “[I felt] not seen at all, so neither heard nor seen, but really treated as if they were treating an underage child.” [Interview 12, Case 25]. In addition, many expressed anxiety and fear regarding their pet’s well-being. One owner recalled: “It drove me crazy not knowing […], what they are doing in there with the [dog].” [Interview 17, Case 35].

#### 3.3.3. Anger, Frustration and Disappointment

Feelings of anger, frustration and disappointment were also frequently mentioned, especially in cases where animals remained untreated despite multiple visits and no adjustment to therapy was made. As one owner put it: “You are so disappointed and […], you no longer have confidence.” [Interview 3, Case 7].

#### 3.3.4. Emotional Stress

Emotional stress following a veterinary error was another recurring theme. Some owners blamed themselves for not acting sooner or for ignoring their instincts. One owner said: “That’s the kind of mistake I also blame myself for, not trusting my own gut feeling.” [Interview 3, Case 7]. Others reported experiencing severe psychological strain.

#### 3.3.5. Lack of Empathy and Connection

Emotions about a lack of trust in the veterinary professionals were also expressed. The quality of the relationship between pet owners and their veterinary surgeons suffered accordingly. A participant said: “I felt that I was not well taken care of there.” [Interview 13, Case 26]. Many described feeling uncomfortable or misunderstood, and the hope of finding someone who would listen and take them seriously was frequently left unfulfilled. This led to disillusionment and further emotional strain.

#### 3.3.6. Conclusion with the Event

In some cases, owners found emotional closure by changing practices, either to find more affordable care or to seek a more trustworthy veterinary surgeon. One owner said: “I was really shocked, and at the same time I was extremely relieved that we had managed to get out of there [the veterinary practice].” [Interview 10, Case 23]. Leaving the original practice behind often marked a turning point in their emotional recovery.

### 3.4. Reactions of the Pet Owners in the Event of an Error

After reflecting on their emotional experiences related to the error, the participants also discussed their immediate reactions when the error occurred. In 27% of the cases, the pet owners remained passive, showing no immediate response to the error and instead adapting to the situation. One participant gave the following reason for not intervening in the situation: “At that moment you are caught unawares.” [Interview 23, Case 45]. On the other hand, 24% of the interviewees decided to change their veterinary surgeon following an error. In 22% of the cases, pet owners contacted the responsible veterinary surgeons, either by returning to the practice with their pets, calling or emailing them, reporting their concerns. Additionally, in 16% of the cases, pet owners reacted instantly and proactively by highlighting the error to the veterinary surgeons. In 11% of the cases, this proactive approach led them to seek help for their animal from emergency veterinary clinics.

### 3.5. Pet Owners’ Perceptions of Handling Errors

Moreover, the views of the pet owners on the conduct of the veterinarians concerning the error were requested. The majority of pet owners (60%) felt that the veterinary surgeon was not aware of the error. Pet owners indicated a lack of accountability (18%) and unprofessional behaviour (18%) often due to pet owners’ perception of neglect of post-anaesthesia care or dismissal of errors with indifference. Additionally, the participants had the impression that some veterinary surgeons responded to errors defensively, justifying their actions or showing annoyance when pet owners raised concerns.

In only 2 out of the 45 cases (4%) did participants feel that those responsible handled the error well by acknowledging it, taking corrective action, reducing costs and apologizing for the incident. Indeed, some veterinary surgeons expressed interest in learning about the diagnosis from the other veterinary practices but failed to acknowledge any errors in their own consultations. One participant reported: “[The veterinary surgeon] acted or reacted well and correctly in my opinion at that moment, because [the veterinary surgeon] said: ‘Oh good that you thought of that, good that you pointed it out to me, of course you’re right.’” [Interview 1, Case 1].

### 3.6. Desired Approach to Errors from the Perspective of Pet Owners

In addition, the pet owners clarified their expectations regarding the handling of the error by veterinary surgeons. In the majority of cases, the pet owners considered it essential that errors are acknowledged, openly communicated and used as a learning opportunity to prevent future mistakes. They expected veterinary surgeons to admit their errors, offer sincere apologies and engage in conversations to analyse and understand the causes of the particular errors. One pet owner said: “If [the veterinary surgeon] had then said, please excuse me, my nerves are on edge and that should not have happened—it won’t happen again, […] but afterwards, back home, we would not have been so upset, and probably would not have made the decision to stop going to the practice.” [Interview 23, Case 45].

Empathy and humane interaction with both pet owners and animals were also highly valued, not only in handling errors but also in daily interactions. Another owner reported: “At the time when [the veterinary surgeon] would have finally done a [consultation], [the veterinary surgeon] might have said OK, I’m sorry that I might not have done it sooner.” [Interview 2, Case 3].

Additionally, professional competence was a key concern, with pet owners expecting veterinary surgeons to provide clear information on treatment methods, risks and alternatives.

They also appreciated honesty when veterinary surgeons recognised their own limitations. One participant reported: “I wish the veterinary surgeon had said: ‘We don’t know what to do next. The dog is getting worse and worse. We recommend discussing the option of euthanasia now or at least considering whether it might be the best.’” [Interview 3, Case 8].

Only in 11% of the cases did participants express a desire for financial compensation, such as discounts or reductions in treatment costs due to errors.

In addition, interviewees emphasised the importance of being presented with multiple treatment options and actively involved in decision-making. Additional concerns included allowing sufficient time for treatment and integrating alternative medicine into conventional veterinary care.

### 3.7. Veterinary Surgeon and Pet Owner Interaction

In 38% of the cases, a conversation about the error took place and in 62% of the cases, no conversation took place. When communication took place, it was most often within three days (53%) or at a later date (29%). In isolated instances it was still pending or held on multiple occasions. Most conversations were conducted with the responsible veterinary surgeon (71%), followed by practice or clinic management (12%) and with other veterinary surgeons in the practice (6%)

A lot of participants reported that they had the impression that the veterinary surgeons did not take responsibility for the error in most cases. A pet owner reported: “[The veterinary surgeon] just dismissed it out of hand.” [Interview 8, Case 20] and another commented: “I just had the feeling that it was more of a defensive wall [...] so they didn’t really let on that an error had been made there.” [Interview 15, Case 33].

In general, most conversations did not help pet owners gain clarity about the cause of the error. Pet owners perceived the error as unacknowledged and that no responsibility had been taken. Additionally, pet owners’ requests, such as wanting their animals to be treated more gently, were not addressed by the veterinary surgeons.

In a few cases, the personal conversation had a positive outcome from the owners’ perspective, leading to reflection and solutions. The pet owners had the impression that the veterinary surgeons responded to pet owners’ fears and complaints, apologised and adjusted the bill. One participant reported: “This [the conversation] helps me a lot in processing the whole event.” [Interview 15, Case 33].

In 28 out of 45 cases no conversation took place, and in 21% of these cases, participants were open to a conversation, while in 46% were not. In 32% of the cases, the question as to whether the pet owners would be open to a conversation was not asked. The most participants who declined a conversation at the time of the interview believed that it would not have been beneficial.

Those who were willing to talk about the error would bring it up with the responsible veterinary surgeons if they were to present themselves again at the clinic. Additionally, now, after the event, a few pet owners stated that they would like to express their dissatisfaction about the error in a personal conversation with the veterinary surgeon.

### 3.8. Awareness and Consequences of Errors

Beyond the emotional impact, the error had tangible consequences. Owner loyalty was lost in 60% of cases, while 22% returned only, when necessary, often for minor treatments such as vaccinations. In 16% the relationship remained unchanged, and in one case a legal dispute ensued. Trust in veterinary surgeons was lost in 62% of cases, limited in 24%, unchanged in 11%, and had never been established in 2%. Following the error, 40% of participants changed their general veterinary practitioner, 27% avoided the institution entirely, and 33% continued without change.

The aftermath of the incident led to reflection on what had happened by some participants and subsequent increased scepticism, mistrust and caution towards veterinary surgeons. One owner said: “I have become more sceptical, more critical.” [Interview 3, Case 7]. After a perceived event of an error, a few pet owners relied more heavily on their instincts, asked more questions, researched treatments or went to specialists. In some cases, the experience also led to feelings of guilt and a sense of responsibility for the error, with owners reproaching themselves for not intervening sooner or not at all. A participant reported: “I believe I suffered harm from the feelings of guilt about having allowed this to happen.” [Interview 14, Case 32]. It was important for three pet owners to share that the event had had a negative impact on their mental well-being.

All participants acknowledged that errors in veterinary medicine can occur. One owner said: “Errors occur—of course they occur.” [Interview 21, Case 37]. It was seen as human, but they had not consciously considered the possibility as such. They expected that an error would not occur with their own pet.

## 4. Discussion

This study is the first qualitative analysis of German pet owners’ perspectives on veterinary errors. Our analysis of 45 reported cases reveals that pet owners valued an acknowledgment of errors and an open dialogue when errors were made. They expected veterinarians to take responsibility, apologise sincerely, and engage in collaborative conversations to prevent recurrence. These insights highlight communication and transparency as critical elements of veterinary education, practice management, and client relations.

### 4.1. Perception of Errors

Most complaints from pet owners concern veterinary errors [36]. However, the term *veterinary error* is inconsistently defined across that study, which limits comparability.

A retrospective analysis of complaints from pet owners in Turkey identified three main categories: clinical, management and relationship issues [10]. Clinical complaints were directly linked to medical errors, particularly in treatment, diagnosis, surgery, medication prescription and anaesthesia [10]. Our findings align with this pattern: owners most often cited errors in treatment, diagnosis, and anaesthesia. Dissatisfaction was particularly strong when ineffective therapies were prescribed without further diagnostic follow-up. The similarity between the Turkish data [10] and our results highlights the need for systematic preventive strategies in daily practice.

Veterinary teams reported similar types of errors. They identified errors in treatment, diagnosis, consultation, medication and anaesthesia [12,37]. In the United States, reported errors were primarily associated with medication and communication, whether at the information source, during transmission or at the point of reception [11].

Taken together, these findings show a consistent picture: complaints and errors cluster around treatment, diagnosis, anaesthesia, and communication [10,11,12,37]. Importantly, both retrospective case analyses and reports from veterinary professionals and pet owners identify the same error domains. This convergence underscores two key implications. First, veterinary teams should treat pet owners’ observations as valuable indicators of error. Second, targeted education and structured prevention strategies are essential to reduce recurring errors and strengthen trust in veterinary practice.

### 4.2. Interaction with Pet Owners

Effective communication with pet owners is a crucial aspect of veterinary practice [38]. Nevertheless, it also carries a certain risk, as it represents a source of error.

Previous studies have shown that inadequate information and unclear decisions account for many complaints, with communication breakdowns repeatedly identified as a major cause [10,39]. Our findings support this, indicating that many errors might have been prevented through clearer and more proactive communication.

Owners in our study consistently reported dissatisfaction when they felt their concerns were dismissed or their pet’s needs overlooked. Loyalty was lost in all such cases, with owners either changing their veterinary surgeon or avoiding the practice altogether. By contrast, when errors were openly acknowledged and addressed in a respectful dialogue, owners valued the accountability shown and were more inclined to maintain trust.

These observations align with wider research showing that satisfaction, compliance and trust are closely tied to effective communication [40,41,42,43]. Loyalty is supported not only by medical competence but also by respectful interaction, transparency and a willingness to acknowledge errors, which foster trust and engagement [40,41,44]. Owners in our study therefore expected to be treated as partners in decision-making, emphasising empathy, honesty and respect alongside professional expertise.

Overall, communication is not an optional complement to clinical skill but a central element of veterinary practice. It shapes daily interactions, determines how errors are managed and directly influences trust and loyalty. Accordingly, structured communication training should be a priority in veterinary education, enabling veterinarians to build resilient and cooperative relationships with clients [38].

### 4.3. Cost Reduction in Case of an Error

Financial implications were secondary for most pet owners interviewed in this study, who prioritised animal care over cost reductions. It was not perceived as important to reduce costs when handling an error. Pet owners see veterinary medicine as a profession where the care of animals takes priority, with financial considerations coming second [24]. On the other hand, veterinary surgeons often feel guilty or unappreciated when discussing costs with pet owners. Consequently, veterinary surgeons should not be discouraged from acknowledging mistakes due to concerns about financial implications. However, they could alleviate financial concerns by carefully analysing client expectations, enhancing communication, offering clear explanations and discussing costs more transparently [24].

### 4.4. Awareness of Errors

Veterinary surgeons often experience negative emotions such as embarrassment and self-doubt, hindering their willingness to discuss mistakes [22]. They avoid reporting errors due to fears of negative consequences, a shortage of opportunities to address them, or the belief that there is no direct impact on patient care. The fear of damaging the client–veterinary surgeon relationship when admitting near misses is another barrier [17].

Our study highlights that pet owners often assume errors go unnoticed. This perception may be influenced by various factors, including a lack of awareness on the part of veterinary surgeons, reluctance to admit errors and inadequate communication. The reasons underlying this perceived lack of awareness were not directly explored in this study which focused solely on pet owners’ perspectives.

Further research could delve into veterinary surgeons’ experiences, identifying obstacles such as fear of repercussions or harm to professional relationships. Understanding these barriers may assist in fostering a more open dialogue between veterinary surgeons and pet owners, ultimately enhancing the recognition and management of errors in veterinary practice.

## 5. Limitations of the Study

The study exclusively focused on cat and dog owners in Germany, limiting its applicability to owners of other animals. Nonetheless, the findings may be relevant to cat and dog owners in other countries with similar human–animal relationships. A notable limitation of our study was the reliance solely on data from pet owners, providing a subjective and one-sided perspective of the situation. No survey was conducted from the perspective of the veterinary professionals to provide a dependent sample. Participants defined *error* without external verification or review to determine whether the care met established medical standards. Consequently, it was not always clear if an event perceived as an error was genuinely one.

The interviewer was conducted by a researcher trained in qualitative methods and supervised by experienced coauthors [23,45,46]. However, it should be noted that she is not a trained psychologist, and other individuals may have obtained different responses.

Data were paraphrased, summarised and analysed by a single primary researcher with supervisory input, which could have led to loss of information, potential unconscious bias and influence from personal experiences. Additionally, the study was conducted virtually, whereas in-person interviews might have gathered more detailed information or revealed different emotional responses [29]. Furthermore, the research was conducted in German, then translated into English, presenting challenges regarding accuracy, consistency and nuances in language.

With only 23 participants involved, the sample size does not allow for broad generalisations about the entire population of pet owners. The recruitment was conducted through social media, potentially skewing the sample towards individuals active on these platforms, excluding others. Also, online interviews might have excluded participants who are less technologically inclined or sceptical of digital communication. Furthermore, the emotional nature of veterinary errors could have deterred some pet owners from participating, thus contributing to a smaller participant pool.

The study revealed a significant gender imbalance, with 74% of the participants being female. This reflects broader patterns of survey participation, where women are generally more likely than men to respond, partly due to social behaviours such as gatekeeping, which can enhance women’s representation [47,48,49,50]. It also corresponds with the fact that 63% of pet owners in Germany were female at the time of the study [51].

It is also worth noting that 30% of the participants in the study had prior medical knowledge, overrepresenting the healthcare sector relative to the general population (13% in Germany) [52,53]. Given that this study overrepresents individuals from the healthcare sector, the findings may not be generalisable to the broader population.

Furthermore, canine owners (76%) were overrepresented compared with feline owners (24%). It is plausible that an increased likelihood of errors may be associated with an increased frequency of veterinary visits, given that dogs are more frequently subjected to veterinary treatment than cats [54].

## 6. Future Research

In future studies, the results of this study should be quantified to obtain a socially representative outcome. It would also be advisable to establish a system that allows pet owners to express their complaints, thus serving as a valuable source of information.

Furthermore, future studies should serve as a basis for the development of guidelines that enable the veterinary team to adopt a professional and constructive manner regarding dealing with errors. In addition, it would be beneficial to incorporate a culture of error into veterinary education, enabling students to learn about and develop good communication skills regarding errors from an early stage.

For future research, it would be beneficial to incorporate the viewpoint of veterinary surgeons and, if required, conduct retrospective analyses of consultations to gain a comprehensive understanding of the scenarios. This would facilitate the identification and analysis of any differences in perception between pet owners and the veterinary team, enabling a comprehensive understanding of the situation.

## 7. Conclusions

This study provides the first qualitative insight into German animal owners’ perceptions of veterinary errors. The owners emphasise the importance of relational aspects, communication, empathy, and acknowledgment, when errors occur. Therefore, it is important to understand the concerns and emotions of animal owners. Our findings underscore that veterinary education and practice should integrate structured error communication to maintain trust and loyalty. By addressing errors transparently and empathetically, veterinary surgeons can support both optimal animal care and strong, trusting relationships with owners, ensuring that clients feel heard, respected, and valued.

Future investigations might explore further dimensions that influence the awareness and reporting of errors in veterinary medicine.

## Data Availability

The data that support the findings of this study are available from the corresponding author upon reasonable request.

## Supplementary Materials

The following supporting information can be downloaded at: https://www.mdpi.com/article/10.3390/ani15202981/s1, Table S1: interview guide.
