# Supplementary material for: German Cat and Dog Owners’ Views on Veterinary Error Handling: Communication and Transparency Concerns from Qualitative Interviews
Source: Animals (Basel). 2025 Oct 15;15(20):2981. doi: 10.3390/ani15202981 (PMC12560880; doi:10.3390/ani15202981)
Supplement: Supplementary file 1 [file animals-15-02981-s001.zip › animals-3901790-supplementary.pdf]

Table S1: interview guide:

1. **General data**
    a. Please tell me your full name, sex and year of birth.
    b. Do you have previous medical knowledge?
2. **Pet owner data**
    a. How long have you had experience as a pet owner?
    b. Do you have a local veterinary surgery?
    c. How do you find a special veterinary surgeon if you have a special problem with your animal?
3. **Personal experience with an error in veterinary medicine**
    a. In which animal species did the event occur?
    b. What exactly happened?
    c. When was the event?
    d. Who made the mistake?
    e. What was the error for you?
    f. How was the error dealt with?
    g. How did you find out about the error?
    h. Who told you about the error?
4. **Personal feelings**
    a. How did you feel in the first moment?
    b. Have your feelings changed over time or during a conversation?
5. **Personal needs**
    a. What do you think they needed at that moment? (Personal need, calm, support, distance)
    b. How did you react to the error? What did you do? (reaction)
    c. What would you have wished for from your veterinary surgeon?
    d. What expectations did you have of the responsible veterinary surgeon in dealing with the event?
    e. How has your relationship with your veterinary surgeon changed because of the event?
    f. How has your trust in your veterinary surgeon changed because of the event?
6. **Conversation**
    a. Were you open to a conversation?
        When?
        With whom?
    b. Are you now open to a conversation?
    c. Was the person in charge ready for a conversation?
    d. Did you feel sufficiently understood and taken seriously during the conversation?
    e. Did the conversation help you to deal with the mistake?
    f. Did this enable you to understand and even accept the error?
    g. Did the conversation help you regain confidence in your veterinary surgeon?
7. **Health education**
    a. Did you feel sufficiently informed about the treatment and alternatives?
    b. Did you feel sufficiently informed about the complications?
    c. Did you feel sufficiently informed about the risks of treatment?
    d. Did you feel sufficiently informed about the costs?
8. **Consequences of the error**
    a. Did you realize beforehand that veterinary surgeons would make mistakes?
    b. Did you change your veterinary surgeon because of the incident?
    c. Do you trust your new veterinary surgeon?

d. Does the event have consequences for you and your animal?

9. **Conclusion**

a. Is there anything else you would like to tell me on this topic?
